# Genetic Control of a Central Pattern Generator: Rhythmic Oromotor Movement in Mice Is Controlled by a Major Locus near *Atp1a2*

**DOI:** 10.1371/journal.pone.0038169

**Published:** 2012-05-31

**Authors:** John D. Boughter, Megan K. Mulligan, Steven J. St. John, Kenichi Tokita, Lu Lu, Detlef H. Heck, Robert W. Williams

**Affiliations:** 1 Department of Anatomy and Neurobiology, University of Tennessee Health Science Center, Memphis, Tennessee, United States of America; 2 Department of Psychology, Rollins College, Winter Park, Florida, United States of America; Barnard College, Columbia University, United States of America

## Abstract

Fluid licking in mice is a rhythmic behavior that is controlled by a central pattern generator (CPG) located in a complex of brainstem nuclei. C57BL/6J (B6) and DBA/2J (D2) strains differ significantly in water-restricted licking, with a highly heritable difference in rates (h^2^≥0.62) and a corresponding 20% difference in interlick interval (mean ± SEM = 116.3±1 vs 95.4±1.1 ms). We systematically quantified motor output in these strains, their F_1_ hybrids, and a set of 64 BXD progeny strains. The mean primary interlick interval (MPI) varied continuously among progeny strains. We detected a significant quantitative trait locus (QTL) for a CPG controlling lick rate on Chr 1 (*Lick1*), and a suggestive locus on Chr 10 (*Lick10*). Linkage was verified by testing of B6.D2-1D congenic stock in which a segment of Chr 1 of the D2 strain was introgressed onto the B6 parent. The *Lick1* interval on distal Chr 1 contains several strong candidate genes. One of these is a sodium/potassium pump subunit (*Atp1a2*) with widespread expression in astrocytes, as well as in a restricted population of neurons. Both this subunit and the entire Na^+^/K^+^-ATPase molecule have been implicated in rhythmogenesis for respiration and locomotion. Sequence variants in or near *Apt1a2* strongly modulate expression of the cognate mRNA in multiple brain regions. This gene region has recently been sequenced exhaustively and we have cataloged over 300 non-coding and synonymous mutations segregating among BXD strains, one or more of which is likely to contribute to differences in central pattern generator tempo.

## Introduction

Rhythmic movements such as those controlling circulatory function, respiration, and locomotion, are fundamental for life. So are rhythmic oromotor movements involved in feeding and drinking, including mastication, fluid licking or lapping, suckling, and swallowing. Like respiration and locomotion, these movements are thought to be controlled by one or more “central pattern generators” (CPGs), intrinsic neuronal circuits in the brainstem or spinal cord that produce rhythmic output [1], [2], [3], [4], [5], [6], [7]. In rodents, licking from a tube may be thought of as a surrogate for a more natural lapping behavior (drinking from an open surface of water), although the tongue movements are not exactly the same [8]. Regardless, licking from a spout has long been used as a model of rhythmic behavior in rats and mice, as it is relatively easy to measure and quantify [8], [9], [10], [11], [12]. Licking involves a pattern of alternating tongue protrusion and retraction, predominantly controlled by extrinsic tongue muscles. Physiological and anatomical evidence suggests that the lick CPG is located in the medullary reticular formation, which either directly or indirectly provides input to the adjacent hypoglossal nucleus [13], [14], [15]. However, the specific neurons, their connectivity, and mechanisms responsible for licking pattern generation are unknown.

Recent studies in mice and other species support the utility of molecular and genetic approaches for dissecting the organization of both invertebrate and vertebrate CPGs [16], [17]. Substantial differences exist among common inbred strains of mice regarding lick rate or speed [18], [19], [20], providing a useful starting point for genetic analysis. Results from two groups using different types of lickometers indicated that the DBA/2J (D2) strain possesses a faster lick rate than C57BL/6J (B6), measured either by lick counts in short (5 s) trials or by computing inter-lick interval (ILI) duration [21], [22]. These strains are the progenitors of the BXD recombinant inbred strain set. This genetic reference population, completely genotyped and currently consisting of ∼150 lines, has been used recently for mapping quantitative trait loci (QTLs) that influence a host of behavioral, anatomical, and physiological phenotypes [23], [24], [25], [26].

In this experiment, we measured fluid licking in 63 BXD strains, as well as B6, D2, and F_1_ mice, of both water and 0.1 M sucrose using a relatively simple, high-throughput licking assay [21]. We sought to identify QTLs that influence licking behavior, especially lick rate and its underlying CPG. Water and sucrose were used in order to investigate the relationship of lick rate to appetitive behavior, with water acting as a “neutral” stimulus relative to sucrose, which is highly preferred to water even in fluid-restricted mice [18].

## Methods

### Animals

Data were collected from adult male and female mice (Mus musculus) from the following strains: B6 (n = 13), D2 (n = 14), B6D2 F1 hybrids (n = 13), as well as two congenic lines: B6.D2 1D (n = 14) and B6.D2 10M (n = 12). We also phenotyped a set of 64 BXD strains (n = 423 individuals). The majority of mice were bred at UTHSC, although some were obtained directly from the Jackson Laboratory (Bar Harbor, ME, USA); these were allowed to acclimatize to the UTHSC vivarium for several weeks prior to testing. B6.D2 1D and 10M congenic mice were offspring of parents kindly provided by Dr Richard Davis and A. Jake Lusis (UCLA); [27]. For B6.D2 1D, the introgressed fragment (from D2 onto the B6 background) spanned from proximal marker rs6267646 (154.395967 Mb) to distal marker rs29609526 (197.134686 Mb). For B6.D2 10M, the introgressed fragment spanned from proximal marker D10Mit299 (66.154402 Mb) to distal marker rs3706484 (114.541067 Mb; locations from NCBI37/mm9).

For BXD strains, the number of cases per strain ranged from 3 to 10, with an average of 6.6, although 5 or more mice were tested in 55 of the 64 strains. Twenty-six of the BXD strains (BXD1 through BXD42) belong to the original sets generated by Taylor and colleagues [28]. The remaining 38 strains were generated by Williams, Lu, and colleagues [29]; www.GeneNetwork.org.

Of the 489 total mice tested, 256 were female and 233 were male. With the exception of 9 of the 64 BXD lines, for which only one sex was tested, males and females were approximately equally represented in each genotype. Previously, we found no difference between sexes in lick rate in relatively large samples of both B6 and D2 mice [21]. The ages of all mice tested in this study ranged from 50 to 381 days, with a mean age of 120.3 days, and a median of 112 days. 85% of the mice tested were between 71–184 days old. Few studies have examined the effects of age on licking or taste: Zhang et al. [30] found no change in lick rhythm in rats from 6 to 12 months of age, but described a slowing of rhythm by 18 months (548 days). In our dataset, age did not co-vary with lick rate across all individual mice (*r* = 0.05). Where possible, we examined age as a factor within strain in our dataset, but did not see effects on lick rate (although the small sample sizes precluded meaningful statistical comparison). In regards to taste, there is little evidence of effects of age on solution preference [31].

Prior to testing, all mice were housed in plastic home cages in a temperature and humidity-controlled vivarium on a 12∶12-h light-dark cycle. Food (22/5 rodent diet, Harland Teklad, Madison, WI) and water were available ad libitum. Approximately 23 h prior to testing in the lickometer, water bottles were removed from the cages of individually housed mice and fresh bedding was provided. Thereafter during the experiment, fluid was only available during daily lickometer tests, whereas food was available (in the home cage) only on an ad-lib basis. Mice were weighed daily prior to testing. The Animal Care and Use Committee at UTHSC approved this study, and all experiments were carried out in accordance with the National Institutes of Health Guide for Care and Use of Laboratory Animals (NIH Publications No. 80–23), revised 1996.

### Apparatus

Mice were tested in the Davis MS160 lickometer (DiLog instruments, Tallahassee, FL, USA). This apparatus consists of an opaque test chamber (28×17.5×16 cm) with a stainless steel mesh floor. Access to a stainless steel sipper tube (aperture = 2 mm) was controlled via a motorized shutter. A test period began when the shutter opened to allow access to the sipper tube, and the mouse made contact with the sipper tube. Lick contact with the sipper tube completed an imperceptible electrical circuit (<50 nA) that allowed the precise time of each contact to be recorded to a computer file to the nearest ms. Each test period ended 20 minutes after the first lick.

### Lick Testing

All mice were tested in the MS-160 for three consecutive days: The mice received deionized water on days 1 and 2, and a 0.1 M sucrose solution on day 3, which is highly palatable to B6 and D2 mice. If a mouse only licked a few times or not at all during the first day of testing, it was retested on the same day, after other mice had been tested. Day 1 was therefore treated as a training day. Lick counts were less robust on this day, with 16% of mice licking less than 100 times. All mice licked ≥123 times on test day 2 (water), and with the exception of a single BXD 25 mouse (50 licks), all mice licked ≥198 times on test day 3 (sucrose). On average, mice maintained 90% of their pre-test body weight on test day 1, 84% on test day 2, and 82% on test day 3 (note that body weights were always sampled prior to fluid access and thus represent the daily minimum). We showed previously that decreasing % body weight as a result of prolonged restricted fluid access results in elevation in the number of licks emitted during a test session, but does not affect lick rate [21].

### Analysis

Total lick counts and Inter-lick ILI durations were analyzed using custom software written by S.J.S. ILI frequency distributions were created for each mouse for each test session. From these distributions, an average measure of ILI duration was derived: The mean primary interlick interval (MPI). This is defined as the mean ILI from the primary component (50–160 ms) of the ILI distribution. As most ILIs fall within this primary component, a smaller MPI value corresponds to a faster lick rate, previously confirmed by counting licks in short trials [21]. Fluid consumption during the test sessions was measured by weighing drinking tubes before and after the session, and volume consumed was corrected for fluid spillage due to bottle handling. Volume per lick (VPL) was derived by dividing the corrected volume by total licks. 19 / 926 VPL values (8 for water and 11 for sucrose) were rejected due to what was likely intake measurement error, e.g. excessive spillage when removing bottles for weighing, or incorrectly recording the bottle weight. The criterion for rejection was +/−3 SD from the mean. Additionally, a sucrose MPI value for one mouse was not calculated due to a corrupted file.

### Statistical analyses

Data were analyzed using a general linear model. Simple regression using a whole model *r* was used to test the influence of factors that may affect the trait under consideration, such as the potential effect of body weight on lick rate or lick count. Effect size (η^2^) was computed as the sums of squares explained by the independent variable over the total sums of squares (in this case, after the sums of squares due to individual subjects has been removed). Broad-sense heritability was estimated in BXD mice by comparing between-strain and total differences using the method outlined by Hegmann and Possidente [32], in which h^2^ = V_A_/(V_A_+2V_E_), where V_A_ = genetic variance and V_E_ = environmental variance. Multiple comparisons among strains, where appropriate, were made using a bonferroni corrected t-test (α/n, where α = 0.05 and n = number of comparisons).

### QTL mapping

All QTL mapping for MPI, total licks, and VPL (both for water and sucrose trials) was conducted using interval-mapping software and genotypes in GeneNetwork.org. We report loci achieving genome-wide significance (p<0.05) and those considered suggestive (p<0.63) based on permutation tests. Linkage was reported in terms of the likelihood ratio statistic (LRS). Bootstrap resampling was used to evaluate the approximate confidence intervals of QTL peaks. All mean strain data are publicly available at GeneNetwork.org (GN identifiers GN12296, 12297, 12601,12602,12604,12605).

### Analyses of gene expression and sequence variants

Multiple expression datasets from multiple brain regions of inbred and BXD strains were used in this analysis (see results). Additional detailed descriptions of strain, sex, tissue preparation, and microarray method for each individual database is available at www.genenetwork.org. Tools for correlating phenotype with expression data are likewise available at this site. RNA–Seq (RNA sequencing) data was generated at UTHSC [33], and is available at http://ucscbrowser.genenetwork.org. Short sequence reads were analyzed using Applied Biosystems whole transcriptome software tools (www.solidsoftwaretools.com/). Reads were mapped to the B6 reference genome (mm9, US National Center for Biotechnology Information (NCBI) build 37) with a minimum alignment score of 24.

## Results

### Strain values for licking and lick rate in inbred and BXD strains

**Figure 1:**
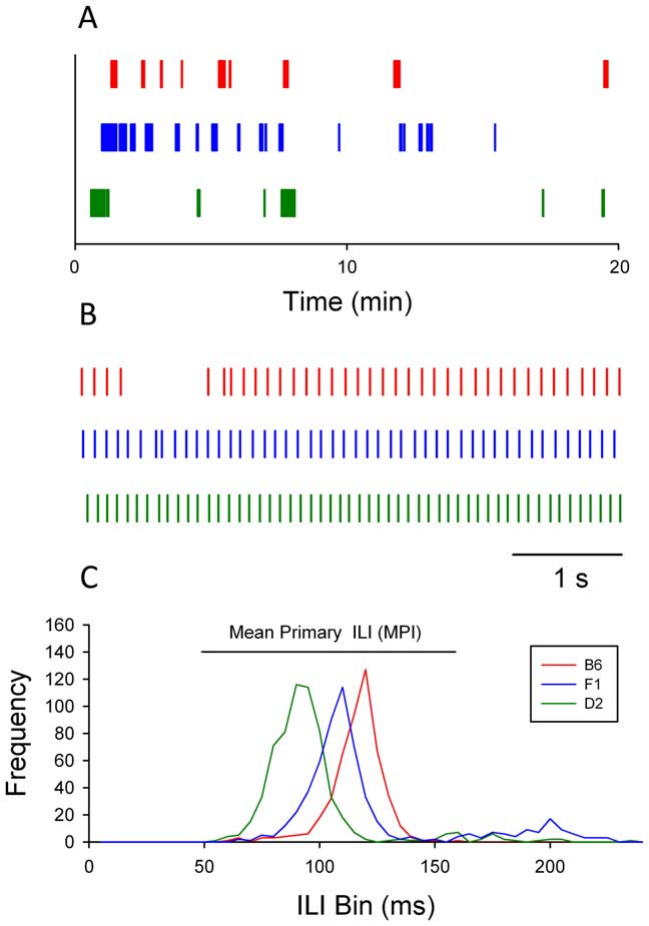
Patterns of licking in representative individual mice. A. In a 20 minute session licks for B6 (red ticks), F_1_ (blue ticks), and D2 (green ticks) mice are organized into a number of drinking bouts; in this view, individual licks are not discernable. B. Expanded view of five seconds of licking for each of the mice shown in A. Each tick denotes a single lick contact C. Frequency distributions of ILIs (bin size = 5 ms)<250 ms for each of these mice show primary distributions with peaks in the range of 50–160 ms. The mean ILI duration in this interval for each mouse is calculated as the MPI (mean primary ILI).

Lick data and ILI distributions from representative individuals illustrate the highly significant and heritable differences between parental strains and the intermediate phenotypes of F1 hybrids (Figure 1). These data were collected on day 2, with water as the stimulus. It is evident that licking across the 20-minute session is organized into discrete bursts of drinking behavior (Fig. 1A), including trains of highly rhythmic licks (Fig. 1B). Inter-lick interval distributions reveal that most intervals occur in a “primary” interval from about 50–160 ms. The mean ILI duration from the primary interval, termed “MPI”, was computed for each mouse on each day (Fig. 1C) [21]. Moreover, it is evident in Figure 1 that the distribution of ILI lengths varies significantly among these strains, with MPI for D2<F_1_<B6.

**Figure 2:**
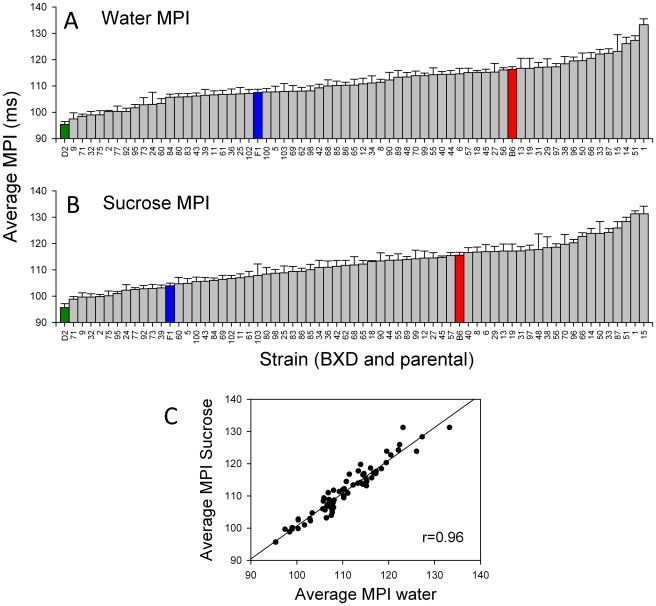
Histograms for average (± SE) mean primary inter-lick interval (MPI) across all BXD, parental, and F_1_ strains. A. Water MPI. B. Sucrose MPI. Green, blue and red bars denote values for D2, F_1_ and B6, respectively. C. Scatter plot of strain averages for water MPI and sucrose MPI.

The distribution of MPIs for both water and sucrose among all strains has a continuous distribution from just below 100 ms to just over 130 ms (Figure 2). The D2 parent is at the fast extreme (lowest MPI) whereas B6 has a higher MPI (corresponds to slower licking). The distribution of strain means did not significantly deviate from normality, for either water or sucrose (Kolmogorov–Smirnov test, p>0.2). Average strain MPI values in both test sessions were highly correlated (Fig. 2C; *r* = 0.96, p<0.05). Repeated measures ANOVA indicated a slight yet significant elevation in MPI from water (mean ± SEM across all mice = 110.39 ms±0.41) to sucrose (mean = 111.05 ms±0.43; F_ [1,395]_ = 10.9; p<0.001). However, the small effect size (η^2^ = 0.027; i.e. less than 3% of the variability in MPI is accounted for by the stimulus) suggests that this effect, while significant, is quite small in magnitude.

**Figure 3:**
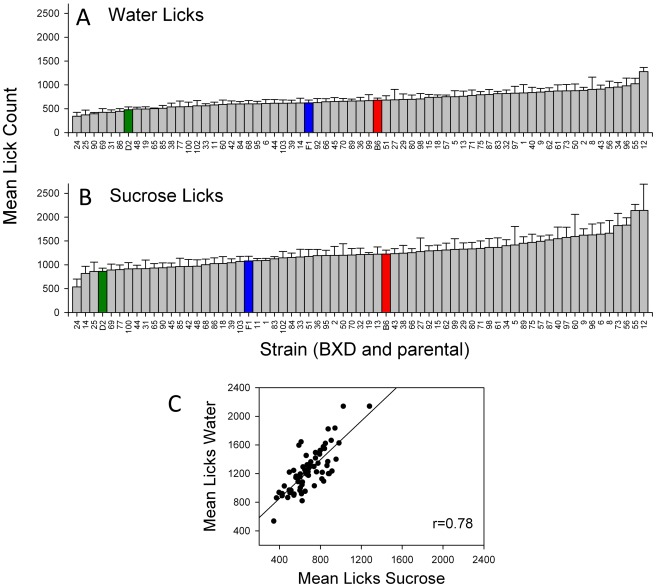
Histograms for mean (± SE) number of licks across all BXD, parental, and F_1_ strains. A. Licks to water. B. Licks to sucrose. Green, blue, and red bars denote values for D2, F_1_ and B6, respectively. C. Scatter plot of strain averages for water MPI and sucrose MPI.

**Figure 4:**
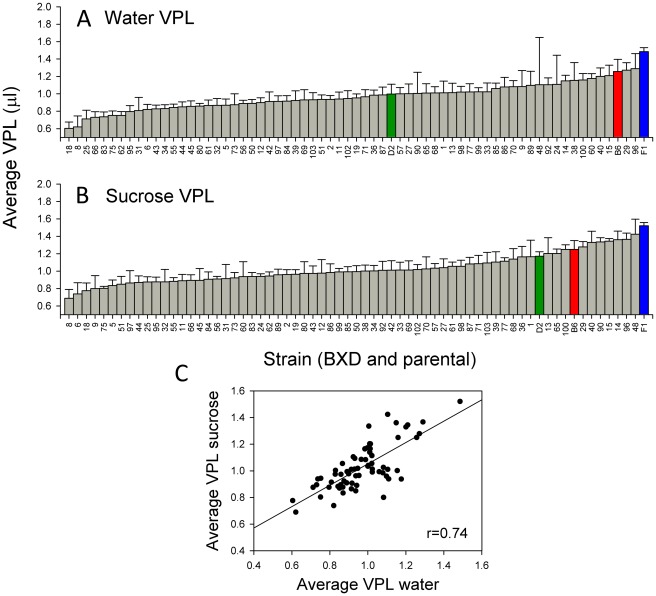
Histograms for average (± SE) volume per lick (VPL) across all BXD, parental, and F_1_ strains. A. Water VPL. B. Sucrose VPL. Green, blue, and red bars denote values for D2, F_1_ and B6, respectively. C. Scatter plot of strain averages for water MPI and sucrose MPI.

Strain distributions were also constructed for either total licks (Figure 3) or VPL (Figure 4) in response to either stimulus. Again, a continuous, normal distribution was found for these variables (K-S, p>0.2). For total licks, mice of all strains possessed higher lick counts in response to sucrose (overall mean ± SEM = 1252.9±23.9) as compared to water (overall mean = 692.6±13.5). D2 mice were on the low end of the strain distribution for total licks (Fig. 3A–B), whereas B6 mice possessed an intermediate phenotype. F_1_ mice were located intermediate to the parental strains. Mean strain values for licks to either stimulus were strongly correlated (*r* = 0.78, p<0.05; Fig. 3C). B6 and F_1_ mice were in the higher end of the range for water VPL whereas D2 mice were intermediate; D2 moved towards the higher end of the range for sucrose (Fig. 4A–B). Interestingly, F_1_ mice possessed the highest VPL for both water and sucrose. While mean strain values for VPL were strongly correlated in both sessions (*r* = 0.74, p<0.05; Fig. 4C), mice of all strains possessed a slightly overall higher VPL for sucrose (mean ± SEM = 1.03±0.01) than for water (0.97±0.01). This difference was significant (F _[1,379]_ = 18.96; p<0.0001).

We examined potential effects of sex on each of these variables across BXD and parental and F_1_ strains. There were no significant effects of sex on MPI for either water (average MPI ± SEM = 110.29±0.56 for females, 110.47±0.61 for males) or sucrose (110.68±0.58 for females, 111.42±0.64 for males). Males of all strains tended to have higher lick counts for both water (mean licks ± SEM = 680.36±18.9 for females, 705.73±19.4 for males) and sucrose (1212.63±30.7 for females, 1297.55±37.1 for males); only the latter was significant (F _[1,393]_ = 4.35; p<0.04). There were no significant effects of sex on VPL.

Correlation matrices (Pearson's *r*) were constructed across all individual cases for each variable (MPI, total licks, VPL, body weight; Table 1) as well as strain means. MPI was not significantly correlated with any other factor for either water or sucrose. Number of licks and VPL were negatively correlated for water and sucrose (*r* = −0.21 for water, and −0.35 for sucrose; p<0.05). This correlation was also evident when strain means were used instead of individual values (*r* = −0.19 for water, and −0.26 for sucrose; only the latter was significant at p<0.05). For water, VPL was also correlated with body weight (*r* = 0.13; p<0.05). These correlations suggest that at least some of the variation in lick count is linked to VPL (i.e. smaller VPL, greater number of licks), and that some of the variation in VPL may be linked to body weight (i.e. smaller mice may possess smaller tongues and hence smaller VPLs).

**Table 1:** Correlations among all individual cases (BXD, B6, D2 and F1 mice^t^) for MPI, total licks, VPL, and body weight.

A. Water				
	MPI	Licks	VPL	Body Weight
MPI	1			
Licks	0.05	1		
VPL	0.05	−0.21*	1	
Body weight	−0.04	−0.04	0.13*	1

B. 01 M Sucrose				
	MPI	Licks	VPL	Body Weight
MPI	1			
Licks	0.09	1		
VPL	−0.01	−0.35*	1	
Body weight	−0.03	−0.04	0.04	1

t n = 455 for water, 451 for sucrose (casewise deletion of missing data).

* p value<0.05.

### Heritability

Broad-sense heritability (h^2^) was calculated using raw data for MPI, total licks, and VPL for both water and sucrose (Table 2). Heritability was strongest for MPI: h^2^ = 0.62 for water and 0.65 for sucrose. The main utility of these estimates is to gauge the likelihood that subsequent mapping studies will yield QTLs. For a behavioral trait, these particular heritability values for MPI are unusually high, a fact that may reflect tight genetic control of brainstem CPGs.

**Table 2:** Estimated broad-sense heritability.

Trait	Heritability
MPI water	0.62
MPI sucrose	0.66
Licks water	0.23
Licks sucrose	0.22
VPI water	0.13
VPI sucrose	0.17

### QTL mapping and validation

**Figure 5:**
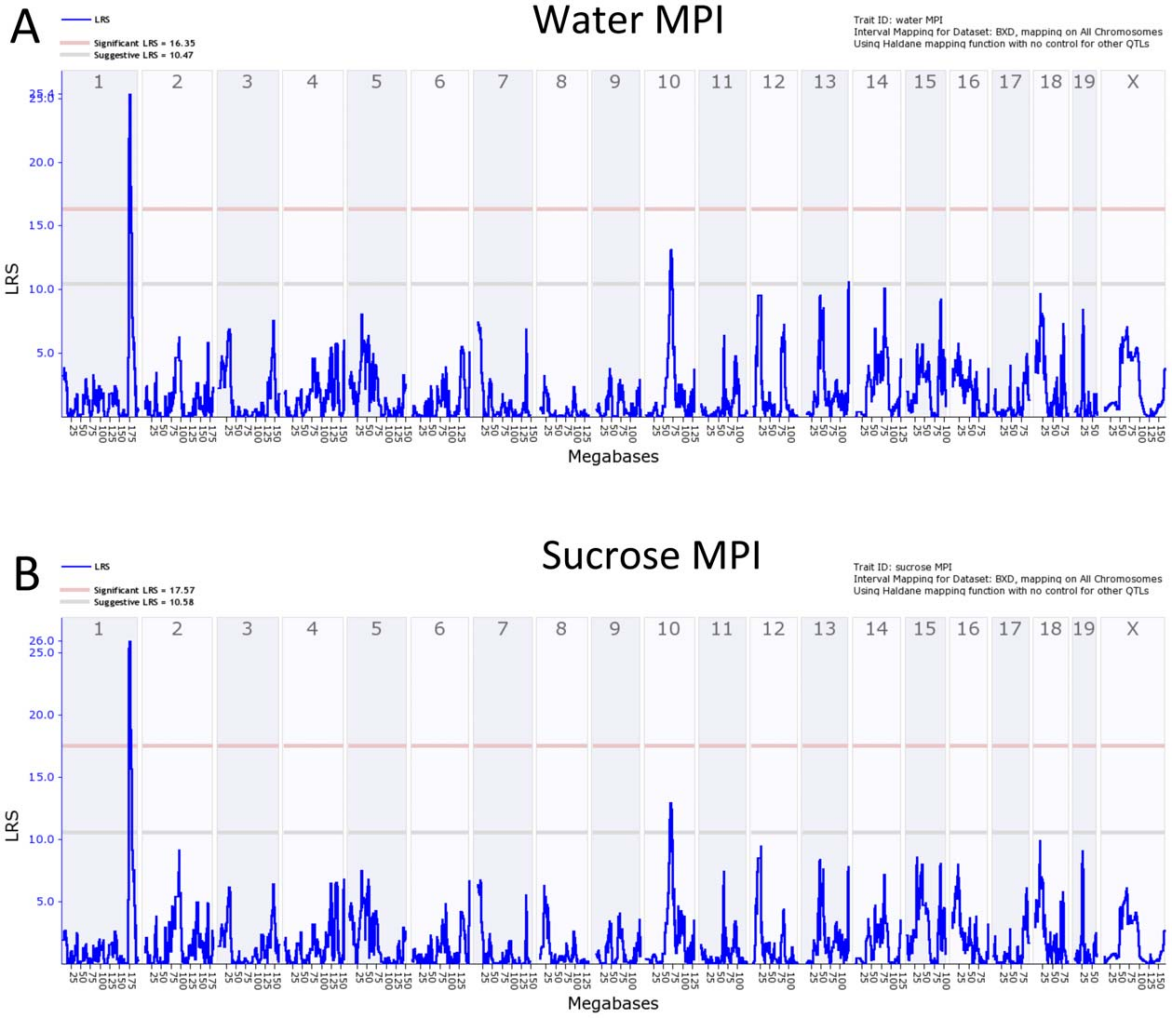
Genome-wide linkage maps of MPI. A. Water MPI. B. Sucrose MPI. Blue trace denotes likelihood ratio (LRS) statistic. A QTL on chromosome 1 exceeds the significance threshold (pink horizontal line; p<0.05) for either stimulus. A suggestive QTL (gray line; p<0.63) was found on chromosome 10.

**Figure 6:**
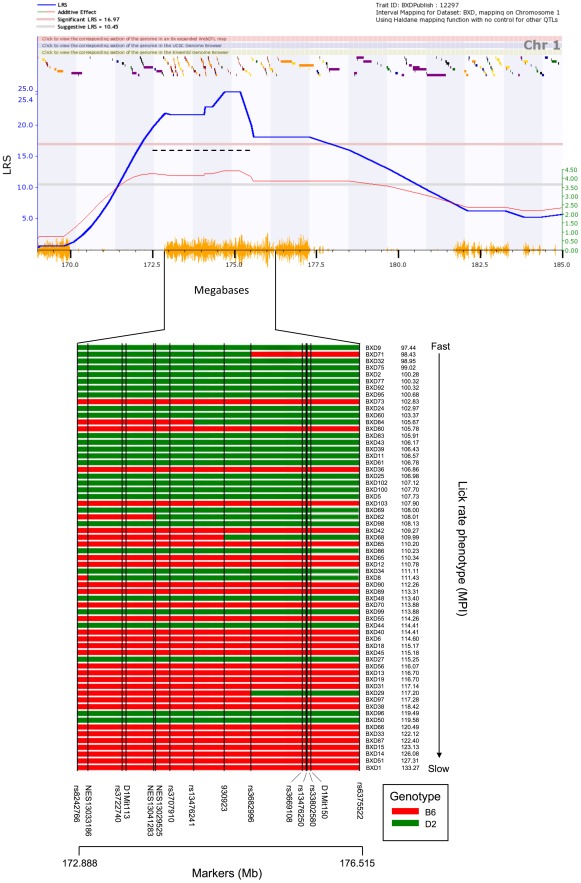
Interval QTL map on part of chromosome 1 for water MPI. Blue trace denotes LRS score, and red trace shows additive effect of the B6 allele. Values for the additive effect are shown in green at right. The LRS score exceeds the threshold for genome-wide significance (pink horizontal line; p<0.05). The dashed black line indicates the confidence interval of the QTL, defined by a 1.5 LOD drop on either side of the peak. Orange seismograph marks indicate SNP density, and colored ticks at top of plot show positions of known genes. Below plot, haplotype distribution among BXD strains is shown for part of the peak QTL region, between the SNP markers rs8242766 and rs6375522. D2 (*D*/*D*) genotype is indicated by green bars and B6 (*B*/*B*) by red bars. Strains in this table are ordered according to MPI score, from fast to slow (top to bottom). The D2 genotype is associated with faster lick rates, as reflected in low MPI values in strains possessing this genotype in the QTL region.

We mapped a strong common QTL for water and sucrose MPI to distal Chr 1 (Figure 5). The QTL for water has a peak likelihood ratio statistic (LRS) of 25.2, equivalent to a LOD (logarithm of the odds) score of 5.5 (Figure 6). The empirical p value of detecting a QTL with this LRS score was determined by mapping 10,000 random permutations of the original data set. For both traits an LRS of 25 has a genome-wide significance level of p<0.001—a highly significant threshold. High MPI values (slow lick rates) are associated with the *B* allele inherited from the B6 parent. The additive effect of the *B* allele at this locus (rs13476241) on MPI is 4.35 ms. The difference between BXD strains that inherited the *D/D* and *B/B* haplotypes in this interval is approximately 9 ms. The confidence interval of this strong QTL—defined by a 1.5 LOD drop on either side of the peak—extends from 172.5 to 175.5 Mb and encompasses the entire proximal region of the QTL-rich region interval on Chr 1 [34]. The peak LRS is located between markers at 174.7 and 175.2 Mb (Figure 6). The sucrose MPI data generated using the same cases maps to the same interval and has a closely matched peak LRS (26.8), effect size, and position. We refer to this QTL shared by the two related phenotypes as *Lick1*.

**Figure 7:**
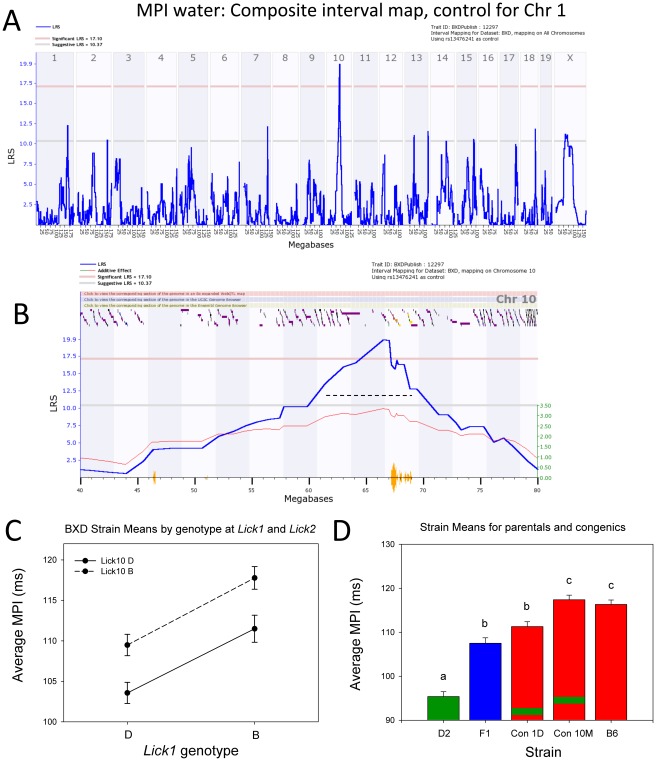
Mapping of a QTL for MPI on Chr 10, and examination of the effects on phenotype of both *Lick1* and *Lick10*. A. Genome-wide Interval map of water MPI, after controlling for the effect of *Lick1* (composite interval mapping). The blue LRS trace shows a significant QTL (pink horizontal line; p<0.05) on Chr 10. B. Enhanced view of QTL (blue trace) on Chr 10 following composite interval mapping. The dashed black line indicates the confidence interval of the QTL, defined by a 1.5 LOD drop on either side of the peak. The red trace shows additive effects of the B6 allele, and the orange seismograph marks indicate SNP density. Additive values are shown in green on right. Colored ticks at top of map indicate positions of known genes. C. Epistasis plot comparing water MPI among BXD strains (means; *n* = 13–19 strains per diplotype at *Lick1* and *Lick10*). The nearly parallel lines in this plot are most consistent with a strictly additive model. D. Histograms comparing average water MPI in D2, F1, B6.D2.1D, B6.D2.10M, and B6 strains (means of individuals; n = 11–14 per strain). Green stripes superimposed on red bars indicate congenic strains. Groups sharing common letters (a,b,c) do not significantly differ, whereas those with different letters significantly differ. Black bars indicate statistically distinct groups, and asterisks denote significant group differences (p<0.005; Bonferroni-corrected t-test).

We controlled for the effect of *Lick1* using the marker rs13476241 (Chr 1 at 174.698878 Mb) and remapped both phenotypes using composite interval mapping (Figure 7). This procedure is essentially the partial regression of lick rate and MPI after eliminating any genetic variance associated with the distal Chr 1 region. We unmasked a second significant QTL on Chr 10 with a peak LRS of 20.5. This LRS has a genome-wide p of approximately 0.005 based on 10,000 permutations of composite mapping, and is also highly significant. The peak is located close to rs13480629 at 67 Mb. The 1.5 LOD confidence interval extends from 61.5 to 69.0 Mb. This QTL is in a SNP desert in the BXD family (the region is essentially identical by descent), but includes a small island of *B* vs *D* SNPs that extends from 67.5 to 69.0 Mb (Fig. 5b). The effect size of this *Lick10* locus has the same polarity as *Lick1* and each *B* allele increases the MPI by 3.3 ms.

We estimated the joint significance of *Lick1* and *Lick10* and their possible interactions using the DIRECT method of Ljungberg and colleagues [35]. The full QTL model defined as MPI = QTL1+QTL2+Q1XQ2 has a total LRS of 45.3 with highly significant additive contributions (Fig. 7C) from *Lick1* (QTL1) and *Lick10* (QTL2) but no two-way interaction term (LRS of 0.28). However, there may be a third locus on Chr 13 between rs13481782 and rs6196305 that interacts epistatically with *Lick1* to generate especially long MPIs when both loci have the *B* haplotype. This interaction has an LRS of 30.9 and has a genome-wide p<0.1. The suggestive Chr 13 locus is also associated with a small additive effect of +2–3 ms per *B* allele.

*Lick1* and *Lick10* account for 33% and 19% of the total between-strain variance in MPI among the BXD strains. These two loci therefore account for approximately 50% of the genetic variation in lick rate. When BXD strains are grouped according to haplotype at *Lick1* or *Lick10*, those possessing mixed haplotypes are intermediate in phenotype to those possessing either solely D2 or B6 haplotypes (Fig. 7C). Moreover, strain MPI means from the congenic strain B6.D2.1D also possessed an intermediate phenotype, significantly different from D2 and B6, but not F1 mice (Bonferroni corrected t-test; p<0.005). B6.D2.1D congenic mice possess a region of chromosome 1, spanning from approximately 156 MB to 199 MB, from the D2 strain introgressed onto a B6 background (Fig. 7D; Davis et al., 2005). Mice from the congenic strain B6.D2.10M were phenotypically similar to B6 mice, although this result does not necessarily indicate failure to confirm the QTL. The introgressed region on these mice spans from approximately 66 MB to 115 MB on Chr. 10. Although the peak LRS score for MPI on Chr 10 was in this region, the lack of phenotypic difference between B6.D2.10M and B6 nevertheless be in part be explained by a physical location for *Lick10* proximal to this fragment (but still within the confidence interval).

**Figure 8:**
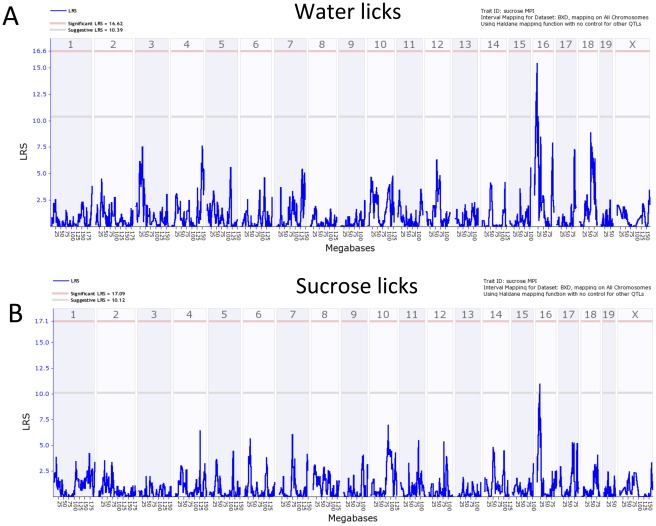
Genome-wide linkage QTL maps of total licks. A. Licks to water. B. Licks to sucrose. A QTL on chromosome 16 exceeds the suggestive threshold (gray line; p<0.63) for either stimulus.

**Figure 9:**
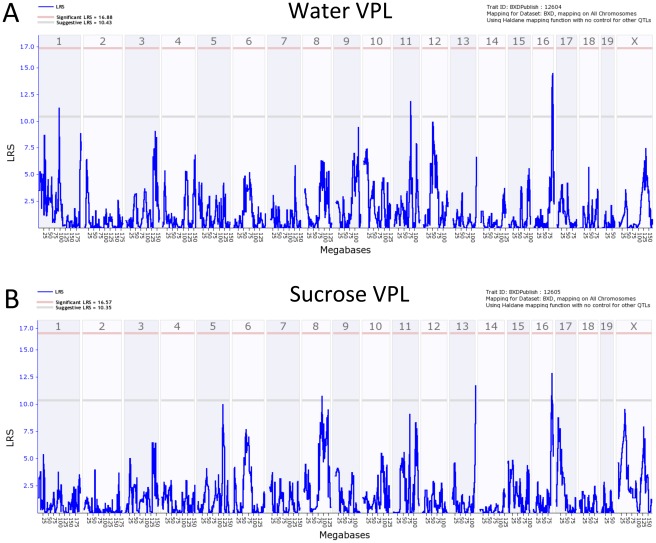
Genome-wide linkage QTL maps of VPL. A. Water VPL. B. Sucrose VPL. QTLs on chromosome 1,11 and 16 exceed the suggestive threshold (gray line; p<0.63) for water. QTLs on chromosome 8,13 and 16 exceed the suggestive threshold for sucrose.

Figure 8 shows interval genome-wide maps for total licks to water (Fig. 8A) or sucrose (Fig. 8B). A suggestive QTL was found for licks to either stimulus on chromosome 16. For VPL for water, suggestive QTLs were found on Chr 1, 11, and 16, and for sucrose VPL suggestive QTLs were found on Chr 8,13, and 16 (Figure 9). The suggestive QTL on Chr 1 for water VPL (peak at approximately 95 MB) did not overlap with the significant lick rate QTL (i.e. confidence intervals do not overlap).

### Candidate Gene Identification

We examined the correlation between strain variation in MPI and the expression of 26 genes in the *Lick1* interval in whole brain tissue [36], and detected strong correlations (*rs*>±0.56, ps<0.002) with three biologically relevant candidate genes—*Atp1a2, Kcnj9, and Kcnj10*. All three are clustered between 174.2 and 174.4 Mb, have high expression in the CNS (including the brainstem), are represented by at least one probe set with a strong cis eQTL and contain multiple B6 and D2 sequence variants (http://ucscbrowser.genenetwork.org).

In order to discriminate among these candidates we performed a partial correlation analysis as described in de la Fuente and colleagues [37] and Mozhui et al. [34]. This procedure removes variance attributable to specific genotype effects on Chr 1 using marker rs13476241 as a controlled factor in a manner similar to composite interval mapping. Of the three candidates, only *Atp1a2* probe sets covaried with MPI (GN 12297) after controlling for genetic variation (Table 3). Residual correlations persist due to underlying biological networks controlled by other sources of variation, such as regulation from other genomic regions and loci.

**Table 3:** Lick1 QTL candidate gene evidence table.

Symbol	Probe Set	Target	Mean	Max Chr1 Marker	cis LRS	Additive Effect	R MPI	Partial R MPI	Partial P MPI
Atp1a2	1455136_at	distal 3′ UTR	10.52	rs3707910	206.66	0.81	−0.54	−0.06	0.66
*Atp1a2*	*1443823_s_at*	*mid distal 3′ UTR*	*13.35*	*rs3722740*	*34.77*	*−0.10*	*0.21*	*−0.20*	*0.15*
*Atp1a2*	*1434893_at*	*Middle 3′ UTR*	*12.51*	*rs3707910*	*93.03*	*−0.27*	*0.44*	*−0.24*	*0.08*
**Atp1a2**	**1427465_at**	**proximal 3′ UTR**	**11.91**	**rs3707910**	**39.12**	**−0.14**	**0.41**	**−0.27**	**0.05**
Atp1a2	1452308_a_at	exons 19 and 20	11.92	rs3707910	5.50	−0.09	0.29	−0.11	0.45
Kcnj10	1419601_at	mid and distal 3′ UTR	10.14	rs3707910	114.45	0.23	−0.47	0.08	0.57
Kcnj9	1428602_at	far 3′ UTR	9.29	rs3707910	33.88	−0.16	0.37	−0.06	0.67
Kcnj9	1450712_at	distal 3′ UTR	9.14	rs3707910	95.18	0.51	−0.45	0.01	0.97
Kcnj9	1426115_a_at	exon 1	7.12	rs13476241	18.88	0.16	−0.26	−0.02	0.90

Mean = Mean expression in the Hippocampus Consortium M430v2 (Jun06) PDNN; R MPI = Pearson correlation between probe set and MPI (record 12297); Partial R MPI = Partial Pearson correlation between probe set and MPI (record 12297) when controlling for marker rs13476241; Partial P MPI = P-value of partial correlation. The additive effect shows the contribution of genotype on average log2 expression. A negative or positive additive effect indicates that the B6 or D2 allele increases trait values, respectively. Probe sets 14348893_at, 1427465_at, and 1452308_a_at have multiple probes that overlap B6 vs. D2 sequence variants and show higher expression associated with the *B* allele which is indicative of a probe artifact (see [78]). However, the use of partial correlation here corrects for these artifacts and any residual covariation is the result of true biological networks. For example, notice the sign change before and after control for these three probe sets to match the sign of the Partial RMPI for the remaining two *Atp1a2* probe sets not affected by probe artifacts.

**Figure 10:**
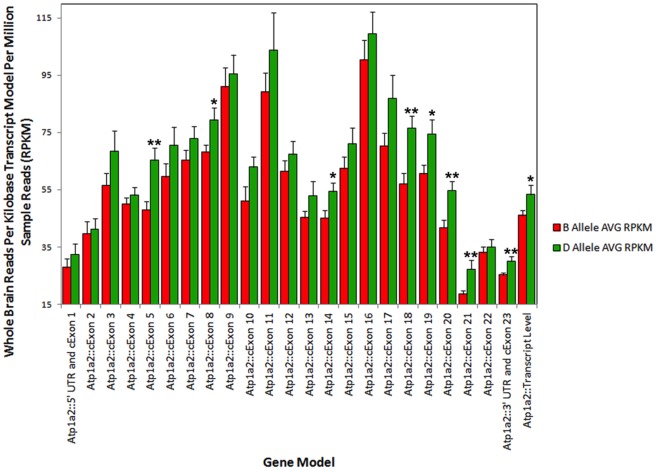
Validation of strain variation in *Atp1a2* expression in the BXD population by RNA-seq. Normalized (RPKM) values in whole brain are shown for the *B* (N = 20) and *D* allele (N = 11) for each feature of *Atp1a2* RefSeq transcript model. “Transcript level” (far right on X-axis) measures expression for the entire *Atp1a2* transcript based on the NCBI RefSeq transcript model. Asterisks denote the level of significance with single and doublets representing p-values less than 0.05 and 0.01, respectively. There is a trend toward higher expression of all transcript features associated with the *D* allele and this difference achieves statistical significance for exons 5, 8, 14, 18, 19, 20, 21, and exon 23 and the 3′ UTR. Consequently, the *D* allele is associated with higher expression of the entire Atp1a2 transcript in whole brain.

A more global analysis of *Atp1a2* expression in brain by RNA-seq of BXD strains confirms higher expression associated with inheritance of the *D* allele (Figure 10). The most striking expression difference occurs for the distal segment of the 3′ UTR, which is reduced 2- to 3-fold in strains with the *B* allele. Higher expression of the distal 3′ UTR in the D2 strain is also confirmed in striatal and eye RNA-seq data (ucscbrowser.genenetwork.org). As suggested by Korostynski et al. (2006) this prominent strain difference in *Atp1a2* expression may be due to alternative 3′ UTR usage [38].

## Discussion

MPI was found to vary among inbred and BXD strains in response to either water or 0.1 M sucrose in a highly heritable manner. Linkage analysis pinpointed a highly significant QTL on distal Chr 1. We earlier reported significant linkage of water MPI to the same region of Chr 1, using both a set of 26 BXD strains (different mice than the current study) and a sample of B6 X D2 F_2_ mice ([39], published abstract; BXD data at genenetwork.org, GN10039). MPI values in the current BXD dataset were highly correlated with values from the earlier study (23 strains in common, *r* = 0.87; p<0.000001). This identity, together with a quantitative effect of distal chromosome 1 substitution through congenic testing, confirms a highly significant QTL controlling lick rate at this location, which we have named *Lick1*. A suggestive QTL for MPI was also found for either stimulus on Chr 10. When we controlled for the effects of *Lick1*, the Chr 10 QTL (*Lick10*) was significant. We also found suggestive linkage on chromosome 16 for total licks – this QTL was found when the stimulus was either water or sucrose (Fig 7), despite the fact that all strains licked more sucrose. The similarity of phenotypes, especially MPI, in response to a neutral (water) or highly appetitive (sucrose) stimulus is consistent with the notion that taste or somatosensory feedback has relatively little effect on lick rate [7], [11], [22], [40].

### Gene candidates for MPI QTLs

Potential neural substrates for the lick CPG are located in the brainstem, including the medulla and pons [7], [14], [41], [42], In general, however, questions remain about which cell types (i.e. interneurons, pre-motor or motor neurons) are critical for various brainstem and spinal cord CPGs, and whether CPG output is driven by individual pacemaker neurons, a synaptically-coupled network, or a combination of these components [1]. Studies with well-characterized invertebrate CPGs suggest a number of likely targets, including ion channels involved in intrinsic neuronal excitability and spiking frequency, as well as elements of synaptic coupling or neuromodulators (for reviews, see [6], [43]. Such gene targets are also suggested by studies of vertebrate CPGs [1], [2], [3], [44]. The QTL on chromosome 1 lies in a gene-rich, well-studied portion of distal chromosome 1, termed *Qrr1*, a QTL hotspot identified by Mozhui et al. [34]. This region is highly enriched in QTLs controlling diverse behavioral and neural phenotypes, as well as a number of regulatory QTLs that modulate expression of a relatively large number of genes [34], [45], [46], [47].

We focused on three positionally and biologically relevant genes located in a short interval from roughly 174.2–174.3 Mb: *Kcnj9*, *Kcnj10*, and *Atp1a2*. *Kcnj9* and *Kcnj10* are cis-regulated potassium channel genes that encode G-protein activated inwardly rectifying potassium channel subunits Kir3.3 and Kir4.1, respectively. Inwardly-rectifying potassium channels have diverse physiological functions, but play an important role in establishing the resting membrane potential of cells, and regulating action potential duration (Hibino et al., 2010). *In situ* expression data from the Allen Brain Atlas (ABA, www.brain-map.org), as well as other expression and immunohistochemical studies, demonstrate that *Kcnj9*/Kir3.3 is expressed strongly in neurons throughout the brain, including in the hippocampus, cerebellum, and brainstem [48], [49], [50]. Variation in *Kcnj9* expression has been strongly implicated in barbiturate withdrawal in mice [51], [52], and possibly in seizure sensitivity [45], [53]. On the other hand, *Kcnj10*/Kir4.1 is expressed diffusely in glial cells [54], [55], [56]. Germaine to a potential link to CPG function is the fact that Kir4.1 is expressed in astrocytes in the ventral respiratory group in the medulla, where it plays a role in K^+^ buffering in the respiratory network. However, ablation of *Kcnj10* did not impair overall respiratory network activity in mice [54].

*Atp1a2* is a cis-regulated gene (Table 3) encoding the α2 subunit of the Na^+^/K^+^-ATPase, located just proximal to *Kcnj9*. Na^+^/K^+^-ATPase (a type of sodium pump) is a membrane protein that plays a critical role in maintaining Na^+^ and K^+^ gradients across the cell membrane. There is strong evidence that Na^+^/K^+^-ATPase plays an important role in rhythmogenesis in the respiration CPG(s) in the brainstem [57], and in the locomotor CPG in the spinal cord [58], [59]. Na^+^/K^+^-ATPase is primarily composed of two major subunits, α and β [60]. The α2-subunit isoform is expressed in a developmental-dependent manner in neurons throughout different regions of the brain, including the ventral respiratory group in the brainstem in E18.5 mice [61], [62]. In the CNS of adult rats or mice it is predominantly and widely expressed in astrocytes, although evidence also suggests localization in a restricted population of neurons, including in hippocampus and spinal cord [61], [62], [63], [64]. Results from brain slice recordings in embryonic *Atp1a*2^−/−^ mice indicate that rhythm generation in respiration-related brainstem neurons is impaired [62], [65]. However, postnatal examination (*Atp1a2* ^−/−^ mice die shortly after birth) suggests normal muscular function [62]. A missense variant in the human *ATP1A2* gene causes Hemiplegia, including numerous motor abnormalities [66], although variations in this gene are not linked to epilepsy [67]. Recent evidence suggests a role for *Atp1a2* in a mouse model of hemiplegic migraine (Cortical spreading depression) [68].

We investigated variants including 24 small insertion/deletions and 288 SNPs located within 1 Kb of the *Atp1a2* locus that are polymorphic between B6 and D2. The majority of these variants are located within introns or intergenic regions. However, 3 insertion/deletions and 41 SNPs are located in the 3′ UTR and 15 SNPs were found in exons; all are silent (synonomous) mutations. Expression of *Atp1a2* is cis-regulated in multiple CNS data sets. Among the BXD family, water MPI is significantly correlated (p<0.00001) with *Atp1a2* expression in whole brain (UCHSC, −0.631; INIA, −0.622; negative correlation indicates higher expression in mice with D2 alleles), cerebellum (GE-NIAA, −0.651), and hippocampus (Hippocampus Consortium, −0.553). Unfortunately, genetic expression data of this type for the brainstem have not yet been generated. Next-generation RNA-seq confirms significantly higher (*p*<0.05) brain expression of *Atp1a2* in strains with the *D* allele at this locus (http://ucscbrowser.genenetwork.org, Figure 10).

How differences in expression of the sodium pump α2 subunit may ultimately correspond to variation in lick rate generation is not entirely clear, but correlates between Na^+^-K^+^ ATPase expression and neuronal function have been described. Anderson et al. [69] isolated the sodium pump via pharmacological block and found a higher density of Na^+^-K^+^ ATPase in fast spiking interneurons (as opposed to pyramidal neurons) and hypothesized that this higher level of activity plays a role in maintenance of high frequency firing rates in this neuron type. Additionally, blocking the pump disrupts rhythmic bursting in rat neonatal spinal cord motor neurons [58]. Alternatively, given the predominance of expression of the a2 subunit in glial cells in the CNS, it may act instead through direct glial-neuron signaling or modulation [70], [71].

The Chr 10 QTL is located in a SNP-poor region, although there is an island of *B* vs *D* SNPs extending from 67.5 to 69.0 Mb (Fig. 5b). Of 15 genes located in this interval, an intriguing candidate for CPG function is *Ank3* (68.99–69.49 Mb) encoding ankyrin G, a scaffolding protein linked to the clustering of voltage-gated Na^+^ channels at the axon initial segment, and required for normal action potential generation and neuronal polarity [72], [73]. *Ank3* is a relatively large gene with multiple splice variants, containing a large number of non-coding synonymous polymorphisms between the B6 and D2 strain. It is strongly expressed in the hypoglossal nucleus in the medulla (Allen Brain Atlas).

Although we focused our search for gene candidates on neuronal/glial cell mechanisms, it is certainly possible that other mechanisms contribute to control of variation in the lick CPG. Anatomical substrates of licking include bone, connective tissue, and oral musculature. Previously, no correlation between tongue length/width or tongue weight with lick rate was found in the parental strains [21]. We conducted a correlation analysis (Pearson's *r*) of MPI data with other published phenotypes (GeneNetwork.org). A study measuring tongue size (Length or weight) in 38 BXD strains did not find linkage to either Chr 1 or Chr 10 [74], and neither variable correlated with MPI (*r*s<±0.03; 44 strains in common). In the Reiner et al. study, tongue weight correlated strongly (*r*s>0.8) with soleus, gastrocnemius, or extensor digitorum (all rear limb muscles) weights [75]. MPI also correlated significantly with several muscle weights (*r*s≤0.58; 20 strains in common). Variation in muscle size or weight may contribute to variation in lick rate, although evidence for this association is indirect. Other physiological or environmental influences may also play a role in producing strain variation. However, studies of both licking and whisking behavior generally demonstrate that although the initiation (and cessation) of these orofacial movements are in part dependent on sensory and motivation-related influences, the underlying rhythm of these movements is relatively independent of such factors [8], [21], [76], [77].

In summary, our work demonstrates strong and precise linkage of variation in lick rate to a gene-rich interval of distal Chr 1 previously implicated in several drug-related and locomotor behaviors. A particularly strong causal candidate in this interval is *Atp1a2*. This gene is expressed throughout the CNS, and has previously been linked to respiratory generation, a closely allied phenotype that is also known to be controlled by brainstem CPGs. The discovery, and eventual confirmation of the contribution of specific genes to natural variation in a CPG-driven behavior such as licking holds great promise for understanding where and how mammalian CPGs are organized in the brainstem and spinal cord.
